# A Model for the Production of Regulatory Grade Viral Hemorrhagic Fever Exposure Stocks: From Field Surveillance to Advanced Characterization of SFTSV

**DOI:** 10.3390/v12090958

**Published:** 2020-08-29

**Authors:** Unai Perez-Sautu, Se Hun Gu, Katie Caviness, Dong Hyun Song, Yu-Jin Kim, Nicholas Di Paola, Daesang Lee, Terry A. Klein, Joseph A. Chitty, Elyse Nagle, Heung-Chul Kim, Sung-Tae Chong, Brett Beitzel, Daniel S. Reyes, Courtney Finch, Russ Byrum, Kurt Cooper, Janie Liang, Jens H. Kuhn, Xiankun Zeng, Kathleen A. Kuehl, Kayla M. Coffin, Jun Liu, Hong Sang Oh, Woong Seog, Byung-Sub Choi, Mariano Sanchez-Lockhart, Gustavo Palacios, Seong Tae Jeong

**Affiliations:** 1 Center for Genome Sciences, United States Army Medical Research Institute of Infectious Diseases (USAMRIID), Fort Detrick, Frederick, MD 21702, USA; upsautu@gmail.com (U.P.-S.); Katie.caviness@gmail.com (K.C.); nicholas.dipaola.ctr@mail.mil (N.D.P.); josephachitty@gmail.com (J.A.C.); elyse.nagle@gmail.com (E.N.); brett.f.beitzel.ctr@mail.mil (B.B.); dsr8721@gmail.com (D.S.R.); 2 The 4th Research & Development Institute, Agency for Defense Development (ADD), Daejeon 34186, Korea; sehungu@add.re.kr (S.H.G.); swpia@add.re.kr (D.H.S.); dslee@add.re.kr (D.L.); 3 Army Headquarters, Gyeryong-si 32800, Korea; vet0721@naver.com (Y.-J.K.); cbs6448@gmail.com (B.-S.C.); 4 Force Health Protection and Preventive Medicine, Medical Department Activity-Korea/65th Medical Brigade, Unit 15281, APO AP 96271, USA; terry.a.klein2.civ@mail.mil (T.A.K.); hungchol.kim2.ln@mail.mil (H.-C.K.); sungtae.chong.ln@mail.mil (S.-T.C.); 5 Integrated Research Facility at Fort Detrick (IRF-Frederick), Division of Clinical Research (DCR), National Institute of Allergy and Infectious Diseases (NIAID), National Institutes of Health (NIH), Fort Detrick, Frederick, MD 21702, USA; courtney.finch@nih.gov (C.F.); byrumr@niaid.nih.gov (R.B.); kurt.cooper@nih.gov (K.C.); janie.liang@nih.gov (J.L.); kuhnjens@mail.nih.gov (J.H.K.); 6 Pathology Division, United States Army Medical Research Institute of Infectious Diseases (USAMRIID), Fort Detrick, Frederick, MD 21702, USA; xiankun.zeng.ctr@mail.mil (X.Z.); kathleen.kuehl@yahoo.com (K.A.K.); kayla.m.coffin2.ctr@mail.mil (K.M.C.); jun.liu2.ctr@mail.mil (J.L.); 7 Armed Forces Medical Command, Seongnam-si 13590, Korea; elvis_presly@naver.com (H.S.O.); afmc23@mnd.go.kr (W.S.); 8 Department of Pathology & Microbiology, University of Nebraska Medical Centre, Omaha, NE 68198, USA

**Keywords:** bandavirus, *Bunyavirales*, bunyavirus, field virologic surveillance, IFNAR^-/-^ mice *Phenuiviridae*, phenuivirus, severe fever with thrombocytopenia syndrome, severe fever with thrombocytopenia syndrome virus, SFTS, SFTSV, target-enrichment high-throughput sequencing, tick borne virus infection, viral hemorrhagic fever, virus exposure stock

## Abstract

Severe fever with thrombocytopenia syndrome virus (SFTSV) is an emerging human pathogen, endemic in areas of China, Japan, and the Korea (KOR). It is primarily transmitted through infected ticks and can cause a severe hemorrhagic fever disease with case fatality rates as high as 30%. Despite its high virulence and increasing prevalence, molecular and functional studies in situ are scarce due to the limited availability of high-titer SFTSV exposure stocks. During the course of field virologic surveillance in 2017, we detected SFTSV in ticks and in a symptomatic soldier in a KOR Army training area. SFTSV was isolated from the ticks producing a high-titer viral exposure stock. Through the use of advanced genomic tools, we present here a complete, in-depth characterization of this viral stock, including a comparison with both the virus in its arthropod source and in the human case, and an in vivo study of its pathogenicity. Thanks to this detailed characterization, this SFTSV viral exposure stock constitutes a quality biological tool for the study of this viral agent and for the development of medical countermeasures, fulfilling the requirements of the main regulatory agencies.

## 1. Introduction

Severe fever with thrombocytopenia syndrome virus (SFTSV; *Bunyavirales*: *Phenuiviridae*: *Dabie bandavirus* [1]) is an emerging tick-borne virus that is distributed throughout much of Eastern Asia and causes severe fever with thrombocytopenia syndrome (SFTS: ICD-11: 1D4E; ICD-10: A93.8) [2]. Clinical signs include acute fever, thrombocytopenia, leukocytopenia, and gastrointestinal symptoms; severe presentations can progress to organ failure and death (12–30% case fatality rate) [3]. The earliest identification and characterization of SFTSV-infected patients and ticks occurred in China in 2010. Thereafter, animal surveillance studies and newfound clinical awareness of SFTS revealed an expansive geographical range and increasing prevalence [4] of SFTSV in Eastern Asia: SFTSV infections have been reported in the Korea [5], Japan, Republic of China/Taiwan, and Vietnam [2,4,6,7,8].

The complete transmission cycle of SFTSV has not been fully demonstrated, although several studies have reported the detection of the virus from ticks—most frequently from Asian longhorned ticks (*Haemaphysalis longicornis*) and also from ticks of other species (e.g., *Amblyomma testudinarium*, *Ixodes nipponensis*, *Haemaphysalis concinna*, *Haemaphysalis flava*, *Rhipicephalus microplus*) [9,10,11,12]. Human-to-human transmission [13] and detection of the virus in different animal species (including goats [14], sheep, cattle, dogs, pigs, chickens [15], cats [16], and rodents [17]) has been documented. While the SFTSV is believed to be primarily transmitted by the bite of infected ticks, secondary transmission has been documented, occurring through contact with body secretions of infected individuals and possibly through contact with infected domestic animal hosts [18,19].

The genome of SFTSV is divided into three single-stranded RNA segments (large [L], medium [M], and small [S]) of negative polarity. The L segment encodes for the RNA-directed RNA polymerase (RdRp), the M segment encodes for the envelope glycoproteins (GPs) GN and GC, and the S segment encodes for both a nucleocapsid protein (N) and a nonstructural protein (NSs) [2,20]. Like other tri-segmented RNA viruses, the evolutionary characteristics of SFTSV make functional genomics studies difficult to execute; high substitution rates, widespread geographical co-circulation between China, Japan, and KOR strains, and the existence of infectious recombinant and reassorted strains [21] complicate genotype-to-phenotype studies. Furthermore, SFTSV is a biosafety level 3 (BSL-3) agent and requires specialized containment facilities and highly trained personnel.

Without a reverse genetics system or well-characterized viral stocks, SFTSV investigations can be limited and slow. Herein, we describe a complete workflow involving intense in-the-field virological surveillance and viral isolation from positive ticks, followed by genomic and phylogenetic characterization of the isolate and analysis of its pathogenic capabilities in an animal model. As a result, we produced a well-characterized, regulatory-grade SFTS virus isolate that can be used under United States Food and Drug Administration (FDA) regulations to test countermeasures and study the different biological aspects of the virus. In addition, we compared the SFTSV from the pool of ticks with SFTSV detected in a human case (death) in which the patient was infected by a tick collected in the same area and time frame. Our work illustrated a comprehensive methodology for the production of quality viral stocks and contributed to our understanding of the population dynamics and the molecular epidemiology of this emergent zoonotic viral pathogen of medical importance.

## 2. Materials and Methods

### 2.1. Human SFTS Case

**Figure 1:**
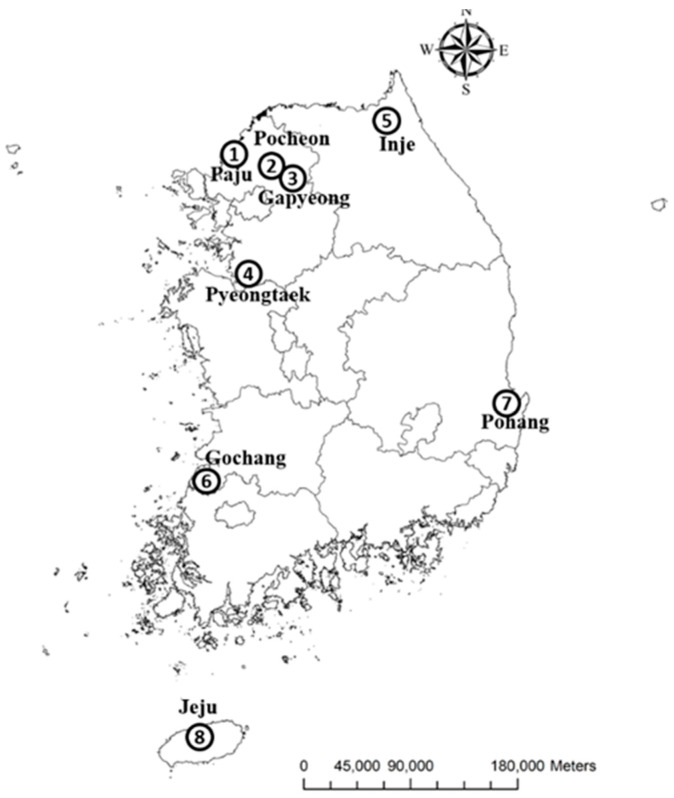
Location of the tick collection points and the severe fever with thrombocytopenia syndrome (SFTS) case. Ticks were collected in: Paju (파주시; 2015; number 1); Jeju Island (제주도; 2015–2016; number 8); Pohang (포항시; 2017; number 7); Gapyeong (가평군; 2017; number 3; severe fever with thrombocytopenia syndrome virus (SFTSV)-positive tick pool); Inje (인제군; 2017; number 5); Pyeongtaek (평택시; 2017; number 4); and Gochang (고창군; 2017; number 6). The SFTS patient (Pocheon [포천시], 2017; number 2) was assigned to the Korea Army (ROKA) base located at Gapyeong (가평군; number 3).

On August 11, 2017, a KOR army soldier (male; 43 years old) assigned to the 8th Division, Korea Army (ROKA), located in Pocheon (포천시, Gyeonggi Province [경기도]) (Figure 1), reported with high fever accompanied by myalgia and chills. The patient was confirmed to be infected with SFTSV by RT-PCR. The patient’s health rapidly deteriorated, with pulmonary edema, sepsis, and multiple-organ dysfunction syndrome (MODS) followed by death on August 24, 2017, after two weeks of hospitalization. Serum samples taken during hospitalization were sent to The 4th Research and Development (R&D) Institute, Agency for Defense Development (ADD) in Daejeon (대전시), KOR, for further investigation. Specimens were stored at −70 °C until processed. The study was approved by the Institutional Review Board (IRB) of the Armed Forces Medical Command (AFMC), KOR (AFMC-19094-IRB-19-070).

### 2.2. Tick Specimens

Three tick surveys were conducted for years 2015, 2016, and 2017, and collected ticks were assayed for the presence of SFTSV by quantitative RT-PCR (RT-qPCR). Ticks (larvae, nymphs, and adults) were collected at different locations in KOR (Figure 1). The 2017 collection point of Gapyeong (가평군; number 3 in Figure 1) was near a training area adjacent to the ROKA military base where the SFTSV-positive ROKA soldier was assigned. Tick surveys were conducted monthly from March to October each year, a time frame that coincided with the primary tick activity period in KOR [22]. Ticks were collected by tick drag [23], placed in cryovials (adults and nymphs alive; larvae in 80% ethanol), returned to the Entomology Section, Force Health Protection & Preventive Medicine (FHP&PM), 65th Medical Brigade, and subsequently identified to the species rank as described previously [5,24,25]. Ticks of the species *H. longicornis*, *H. flava*, *H. phasiana*, *I. niponnensis*, and *R. sanguineus* were pooled by species, stage of development, sampling location, and year of collection (a total of 726 pools) and processed for RT-qPCR analysis (Table 1).

### 2.3. SFTSV Detection by Molecular Assays

SFTSV detection in the ROKA patient was performed by The 4th R&D Institute, ADD, Daejeon (대전시), KOR. Total RNA was extracted from serum specimens of the SFTS patient using the QIAamp Viral RNA Mini Kit (Qiagen, Hilden, Germany) following the manufacturer’s instructions. cDNA was prepared using the SuperScript III First-Strand Synthesis System (Invitrogen, San Diego, CA, USA) and random hexamers. SFTSV was detected by means of a nested PCR with the following oligo-nucleotide primers: SFTSV-S1F (5′-ACACAAAGACCCCCTT-3′) and SFTSV-S406R (5′-CATTGCCCAGCTGTTCT-3′) followed by SFTSV-S1F and SFTSV-S302R (5′-AGTAGCACCTCATGTCC-3′) for the S segment; SFTSV-M86F (5′-CAATCATCTGCGCAGGAC-3′) and SFTSV-M555R (5′-AGCAACATCACCTATCCA-3′) followed by SFTSV-M177F (5′-GCTGATACATGTTTCGTC-3′) and SFTSV-M495R (5′-GTCTGAACTGCAGAGCTC-3′) for the M segment; and SFTSV-L1F (5′-ACACAGAGACGCCCAGAT-3′) and SFTSV-L343R (5′-CATCCCATCAGAACCATCAT-3′) followed by SFTSV-L1F and SFTSV-L263R (5′-TGAAGTCATGATTGATCT-3′) for the L segment. Initial denaturation was at 94 °C for 5 min, followed by 15 cycles of denaturation at 94 °C for 40 sec, annealing at 50 °C for 40 sec, and elongation at 72 °C for 1 min. This was followed by 25 cycles of denaturation at 94 °C for 40 sec, annealing at 52 °C for 40 sec, and elongation at 72 °C for 1 min. PCR was performed on a ProFlex PCR System (Applied Biosystems, Foster City, CA, USA). PCR products were purified with the QIAquick PCR Purification Kit (Qiagen), and DNA sequencing was performed in both directions using the BigDye Terminator v3.1 Cycle Sequencing Kit (Applied Biosystems, Foster City, CA, USA) on a 3500 Series Genetic Analyzer (Applied Biosystems).

SFTSV detection in tick specimens was performed as follows: Tick pools were homogenized in 500 μL of BD Universal Viral Transport Medium (Beckton Dickinson, Franklin Lakes, NJ, USA) using a 1600 MiniG Automated Tissue Homogenizer and Cell Lyser (SPEX SamplePrep, Metuchen, NJ, USA). After homogenization, preparations were visually inspected to verify that ticks were broken down into small pieces. Homogenates were transferred to new Eppendorf tubes, centrifuged for 15 min at 5000× *g* at 4 °C, and supernatants were stored at −80 °C until further use. RNA was extracted from 100-µL aliquots of supernatants with TRIzol LS Reagent and the Phasemaker Tubes Complete System according to the manufacturer’s guidelines (Thermo Fisher Scientific, Waltham, MA, USA). Detection of SFTSV was performed using 5-µL RNA extracts using a multiplex RT-qPCR method, targeting the three genomic segments of the viral genome as previously described [26], with minor modifications introduced in the sequences of the primers and probes (Table 2).

RT-qPCR was performed with the AgPath-ID One-Step RT-PCR kit (Thermo Fisher Scientific) in a CFX96 Touch Real-Time PCR Detection System (Bio-Rad, Hercules, CA, USA). The assay was designed according to the manufacturer’s guidelines and using a final concentration of 0.4 µM for the primers and 0.12 µM for the probes, in total reaction volumes of 25 µL each. Cycling conditions were as follows: 30 min at 50 °C, followed by 10 min at 95 °C, and 35 cycles for 15 sec at 95 °C, and 45 sec at 60 °C, after which fluorescence was measured. For viral RNA quantification, a standard curve was generated against a synthetic oligonucleotide (one for each genomic segment) and expressed as genome copies (c)/mL.

### 2.4. Viral Isolation and Characterization

#### 2.4.1. Viral Isolation and Titration

Detection and isolation of SFTSV from the positive tick pool was performed in the BSL-3 biocontainment laboratory at the U.S. Army Medical Research Institute of Infectious Diseases (USAMRIID) following U.S. Centers for Disease Control and Prevention (CDC) and institutional BSL-3 safety guidelines. Grivet (*Chlorocebus aethiops*) Vero E6 cells (ATCC #CRL-1586) were cultured in Eagle’s Minimum Essential Medium (EMEM, Thermo Fisher Scientific), supplemented with 10% heat-inactivated fetal bovine serum (FBS), 2 mM l-glutamine, 100 U/mL penicillin, and 100 µg/mL streptomycin (Thermo Fisher Scientific). All cells were maintained at 37 °C with 5% CO_2_. To isolate SFTSV from the positive tick pool, the tick homogenate was filtered through a 0.45-µm syringe filter and applied as a low volume (100-µL) inoculum to a single sub-confluent well of a six-well dish of Vero E6 cells. The virus inoculum was incubated at 37 °C for 1 h. Then, 2 mL of fresh medium was added to the well and incubated for 7 d (Passage 1).

Virus or control supernatants and cells were passaged 7 d after infection to yield Passage 2 as follows: Supernatant was collected and clarified by centrifugation at 3000× *g* for 10 min. Fresh medium was added back to the virus-infected or control wells and the cells were scraped, collected in a conical tube, and mechanically homogenized. Medium was removed from two new sub-confluent T-25 flasks, and 1 mL of clarified supernatant or 1 mL of homogenized cells was added to each flask and allowed to incubate at 37 °C. After 30 min, 7 mL of fresh medium was added to each flask and cells were incubated for 7 d.

To yield Passage 3, virus or control supernatants and cells were passaged as described above for Passage 2, except T-75 flasks were used to increase the total virus yield. After Passage 3 was incubated for 7 d, supernatants from SFTSV-containing T-75 flasks were collected, clarified, aliquoted, and stored at −80 °C until use.

Viral stock titration was performed as follows: Passage 3 stocks of SFTSV were titrated using an infectious cell culture assay in Vero E6 cells. Because detectable cytopathic effect (CPE) was not observed during the SFTSV isolation attempts in this cell line, a multiplex RT-qPCR assay was used to estimate the 50% tissue culture infectious dose (TCID_50_). Briefly, Vero E6 cells were plated in 96-well dishes that were expected to be ~80% confluent the next day. Twenty-four hours following plating, the cells were transferred into the BSL-3 laboratory, and the SFTSV stock was serially diluted six independent times. These replicates were used to infect the Vero E6 cells. After 7 d, supernatants were collected, clarified as described above, and inactivated using a 3:1 ratio of TRIzol LS supernatant per manufacturer guidelines. RNA extraction and SFTSV detection by multiplex RT-qPCR were performed as described above. Every dilution from each of the six replicates was assayed in triplicate in the multiplex RT-qPCR, the presence or absence of virus in each dilution and replicate was determined, and the TCID_50_ titers were calculated following the method described by L. J. Reed and H. Muench [27].

#### 2.4.2. Transmission Electron Microscopy

Transmission electron microscopy (TEM) analysis was performed using a method reported previously [28]. Vero E6 cells were plated onto electron microscopy (EM) 35-mm dishes with EM coverslips. The next day, cells were transferred to a BSL-3 laboratory and were mock-exposed or exposed to Passage 3 SFTSV with RT-qPCR cycle thresholds ranging from 13–17. At Day 3 and Day 6 after exposure, medium was removed, and cells were washed with phosphate-buffered saline (PBS, Thermo Fisher Scientific) before the entire dish and lid were submerged in TEM fixative (4% formaldehyde, 2.5% glutaraldehyde, and 0.1 M of sodium cacodylate pH 7.4 buffer, JEOL Ltd., Akishima, Tokyo, Japan). In accordance with standard laboratory BSL-3 biocontainment procedures, one dish and one lid were left completely submerged in TEM fixative for more than 24 h before removal from the BSL-3 laboratory for further processing. After washing three times in 0.1-mM sodium cacodylate buffer at 2.5 °C for 3 min each, the primary-fixed cells were post-fixed by incubation with 1% osmium tetroxide (Millipore Sigma, St. Louis, MO, USA) in 0.1 M of sodium cacodylate for 1 h on ice. After washing with distilled water three times for 3 min each, the fixed cells were stained and stabilized in 1% uranyl acetate (Millipore Sigma) at 2.5 °C for 1 h and dehydrated in a series of 25%, 50%, 75%, and 95% ethanol at 2.5 °C for 3 min each. Cells were then dehydrated at room temperature three times for 3 min each in 100% ethanol and infiltrated with well-mixed 50% ethanol, 50% Durcupan ACM resin (Millipore Sigma) for 1 h with agitation at room temperature, followed by 100% Durcupan ACM resin twice for 3 h with agitation. Infiltrated samples on glass coverslips of the MatTek dishes were placed in a hybridization oven for polymerization at 60 °C for at least 48 h. Glass coverslips were peeled away from the bottom of the MatTek dishes using razor blades and were cut out arbitrarily into small pieces. The pieces were then glued with the cell side up to a block for sectioning. Thin sections (~80 nm thick) were collected and pre-stained with 1% uranyl acetate and Reynolds’s lead [29] before examination using a 1011 Transmission Electron Microscope (JEOL, Akishima, Japan) at 80 kV. Digital images were acquired using the Advanced Microscopy Techniques camera system (Advanced Microscopy Techniques, Danvers, MA, USA).

#### 2.4.3. Immunofluorescence Microscopy

For immunofluorescence microscopy analysis, Vero E6 cells were plated on coverslips of 24-well dishes and incubated overnight at 37 °C. The following day, the cells were transferred to a BSL-3 laboratory and were mock-exposed or exposed to SFTSV from Passage 3 and incubated for 5 d. Following incubation, medium was removed, cells were washed with PBS, and the entire dish and lid were completely submerged in 10% formalin (final concentration: 4% formaldehyde) for at least 24 h. After fixation, dishes were removed from the BSL-3 biocontainment laboratory and stored at 4 °C until further processing. Briefly, after rinses with phosphate-buffered triton (PBT; 0.1% Triton X-100 [Millipore Sigma] in PBS, pH 7.4), the cells were blocked with PBT containing 5% normal goat serum (Thermo Fisher Scientific) overnight at 4 °C and then incubated with rabbit anti-SFTSV polyclonal antibody (pAb; Abnova, Taipei, Republic of China/Taiwan; 1:500) and mouse anti-GM130 pAb (BD Biosciences, Franklin Lakes, NJ, USA; 1:50) for 2 h at room temperature. After rinses with PBT, the sections were incubated with secondary goat anti-rabbit Alexa Fluor 488 antibody (Thermo Fisher Scientific; 1:500) and goat anti-mouse Cy3 pAb (Thermo Fisher Scientific; 1:500) for 1 h at room temperature. Cells were mounted using Vectashield Mounting Medium with DAPI (Vector Laboratories, Burlingame, CA, USA). Images were captured on an LSM 880 confocal system (Zeiss, Oberkochen, Germany) and processed using ImageJ software (National Institutes of Health, https://imagej.nih.gov/ij/). A commercially available SFTSV pAb (Abnova; #PAB27171) raised against the SFTSV HB29 strain was used to detect the SFTSV GPs. An antibody to GM130 (BD Bioscience, Franklin Lakes, NJ, USA; #610822) was used to label the Golgi apparatus.

### 2.5. Target-Enrichment Whole-Genome Sequencing

Viral genomes from the serum samples of the patient, from the RT-qPCR-positive tick pool, and from the SFTSV isolate from Passage 3 were obtained through target-enrichment high-throughput sequencing (HTS). Sequencing libraries were prepared from RNA extracts using a modified version of the Illumina TruSeq RNA Exome Kit (Illumina, San Diego, CA, USA) as previously described [30]. For this study, SFTSV-specific 80mer probes tiled along the three genomic segments of the virus were used for library selection and enrichment. Samples were barcoded with non-overlapping dual indexes in a true dual-indexing configuration and pooled and sequenced using the MiSeq Reagent kit v2 or v3 (Illumina) on a MiSeq instrument (Illumina). Sequencing runs were performed at 2×151-bp reads.

Complete genome consensus sequences were generated from the HTS data as follows: Open-source programs Cutadapt [31], Prinseq-lite [32] and Picard (http://broadinstitute.github.io/picard/) were used for preprocessing of samples, including adapter removal, PCR duplicate removal, and quality filtering of the index (<30 Phred) and reads (<20 Phred). For HTS data obtained from the human case, consensus sequence generation was performed using CLC Genomics Workbench version 7.5.2 (CLC Bio, Cambridge, MA, USA) with SFTSV strain KASJH (GenBank #KP663733, KP663732, and KP663731) as the reference sequence. For HTS data obtained from the tick pool and from the SFTSV cell-culture isolate, a validated analysis pipeline was used [33], with SFTSV strain KACNH3 (GenBank #KP663745, KP663744, and KP663743) as the reference sequence. Briefly, after adapter removal and quality filtering steps as described above, reads were aligned to the reference using Bowtie2 [34], duplicates were removed with Picard, and a new consensus was generated using a combination of Samtools v0.1.18 [35] and custom scripts. Only bases with a Phred quality score ≥20 were utilized in consensus calling, and a minimum of 3× read-depth coverage, in support of the consensus, was required to make a call; positions lacking this depth of coverage were treated as missing (that is, called “N”). Sequence similarity analysis was performed with MUSCLE [36].

### 2.6. Genomic Characterization

In-depth viral population characteristics from the SFTS case, the tick pool sample, and the Vero E6 isolate were studied using a validated analysis pipeline (VSALIGN) [37]. VSALIGN is built on Perl and uses open-source programs Cutadapt [31] and Prinseq-lite [32] for preprocessing of samples, including adapter removal, PCR duplicate removal, and quality filtering of the index (<30 Phred) and reads (<20 Phred). Additional preprocessing steps were included to remove chimeric sequences, single or low quality paired-end reads, and reads that did not have any homology to the reference sequence. Sequences were aligned to the consensus SFTSV genome generated from ticks (reference sequence) using default parameters in VSALIGN to determine the frequency (events per site per genome) of single nucleotide polymorphisms (SNPs), insertions and deletions (indels), transitions, and transversions and to determine subclonal diversity (SCD) measured as the combined frequency (events per site per genome) of the aforementioned parameters. These viral population diversity parameters were measured for nucleotide positions meeting a minimum depth of 100 reads.

### 2.7. Phylogenetic Analysis and Haplotype Network

All publicly available SFTSV segment sequences were downloaded from GenBank through the Viral Pathogen Database and Analysis Resource (www.viprbrc.org) on April 2, 2020. Coding sequences from L (RdRp) and M (GN and GC) segments were extracted; sequences from the S segment were split by NSs and N coding sequences. An in-house script removed all strains that did not have all four coding sequences (https://github.com/rainakumar/common_fasta_utils). Individual coding sequences were concatenated in Geneious version R9 (www.geneious.com) to form coding-complete genomes. Strains with confirmed reassortments were removed from downstream Bayesian analyses [38]. GenBank accession numbers and metadata can be found in Table S1. Concatenated sequences were aligned using MAFFT v7.388 [39] and manually curated in Geneious. A maximum-likelihood tree was inferred using FastTree version 2.1 [40] with a generalized time reversible (GTR) nucleotide substitution model, exhaustive search, pseudotypes, 5000 bootstrap repetitions, and 2000 gamma rate categories (Figure S1).

A Bayesian-inferred, maximum-clade credibility phylogenetic tree and associated evolutionary rates were estimated using the Hasegawa–Kishino–Yano 85 (HKY85) model [41] for 207 concatenated coding sequences and general time-reversible model [42] for 19 genotype B-1 concatenated coding sequences. Both models included gamma-distributed rate variation (+ Γ_4_) and invariant sites (+ I), which were inferred by jModeltest2 [43]. Partitions shared a constant-size coalescence prior and a continuous time Markov chain reference prior [44]. An uncorrelated lognormal relaxed molecular clock was independently inferred (unlinked) across four partitions (L, M, N, and NSs open reading frames [ORFs]). Each analysis consisted of 10 × 10^8^ Markov chain Monte Carlo steps (25% of which were discarded as burn-in); parameters and trees were sampled every 100,000 generations. Tracer v.1.6 [45] was used to ensure run convergence (effective sample size >200), and TreeAnnotator 1.8.4 [46] was used to calculate a maximum-clade credibility (MCC) tree using a posterior probability limit of 0.7.

### 2.8. IFNAR^-/-^ Mice Infections

Adult (7–8 weeks old), interferon (α/β) receptor knockout (IFNAR^-/-^) laboratory mice (strain: B6.129S2-Ifnar1tm1Agt/Mmjax) were purchased from the Jackson Laboratory (Bar Harbor, ME, USA). The mice were acclimated for 6 days to the Maximum Containment (BSL-4) Laboratory within the National Institutes of Health (NIH), National Institute of Allergy and Infectious Diseases (NIAID), Division of Clinical Research (DCR) Integrated Research Facility at Fort Detrick (IRF-Frederick). Animals were housed in sterilized, ventilated cages (One Cage, Lab Products, Seaford, DE, USA). Male mice were housed individually while female mice were housed 3 or 4 per cage on paper bedding (TEK-Fresh 7099, Envigo, Madison, WI, USA) and provided sterilized food (Teklad Global Rodent Chow, 2018SX, Envigo, Madison, WI, USA) and water without restriction. The water was treated by a reverse-osmosis system (RO8600, Avidity Sciences, Waterford, WI, USA) prior to sterilization by autoclaving in bottles. Additional nesting material (Enviro-dri, Shepherd Specialty Papers, Watertown, TN, USA) was added to each cage. Environmental conditions, including air changes, temperature, humidity, and light cycle were controlled. Air changes were 22–24 per hour, temperature was set to 72 °F, humidity was set to 50%, and the light:dark cycle was 12:12 h. All animal manipulations, including cage changes at two-week intervals, were performed in a class II biosafety cabinet within the animal room. The mice were divided into two groups of 13 animals per group (six males/seven females). One group (“virus”) was inoculated subcutaneously (0.2 mL) with 1 × 10^5^ TCID_50_ of SFTSV strain USAMRIID-HLP23_VE6. The other group (“mock”) was inoculated with the same volume of PBS (Gibco, Gaithersburg, MD, USA). Mice were weighed 5 d prior to exposure (Day -5) and daily after inoculation (Day 0–Day 21) and monitored twice daily for signs of disease. All observations were recorded along with the weight measurements. All animal care and experimental procedures were performed in accordance with Comparative Medicine SOPs and the experimental protocol approved by the IRF-Frederick Animal Care and Use Committee.

## 3. Results

### 3.1. Detection of SFTSV in Ticks

A total of 13,636 tick specimens (larvae, nymphs, and adults) were collected and distributed in 726 pools according to species, stage of development, sampling location, and year of collection (Table 1). Larvae (4861 specimens; ~26 specimens per pool) were distributed in 186 pools, nymphs (8509 specimens; ~19 specimens per pool) were distributed in 445 pools, and adults (266 specimens; ~3 specimens per pool) were distributed in 95 pools (Table 1). All 726 pools were analyzed by RT-qPCR for the presence of SFTSV. Only one out of 726 pools (0.14%) was positive for SFTSV by multiplex RT-qPCR assay. This pool (Pool 23) consisted of 59 Asian longhorned tick nymphs collected in 2017 at a military training area adjacent to the ROKA base in Gapyeong (가평군), Gyeonggi Province (경기도), where the SFTSV patient was assigned (Figure 1).

### 3.2. Isolation and Characterization of the SFTS Virus from the Tick Pool

#### 3.2.1. Viral isolation and Titration in Vero E6 Cells

The virus was first isolated from the positive tick pool. Sub-confluent Vero E6 cells were inoculated with the filtered, positive-tick-pool homogenate and incubated for 7 d (Passage 1). Supernatants and Vero E6 cell homogenates were blindly passaged two additional times, each 7 d apart (Passage 2 and Passage 3). As infection of Vero E6 cells with the tick homogenate did not induce CPE in any of the passages, we used multiplex RT-qPCR to confirm the presence of SFTSV. All passages tested positive (results not shown). Passage 3 was additionally quantified with the multiplex RT-qPCR assay, measuring genome segment copy numbers of 5.29 × 10^9^ c/mL, 7.97 × 10^9^ c/mL, and 9.42 × 10^9^ c/mL for the S, M, and L segments, respectively. The infectious virus titer of Passage 3 was determined by a modified TCID_50_ assay, during which multiplex RT-qPCR was used to determine the presence or absence of virus in each dilution and replicate, resulting in a virus titer of 2.51 × 10^7^ TCID_50_/mL.

#### 3.2.2. SFTSV Particles Co-Located with the Golgi Apparatus in Vero E6 Cells

**Figure 2:**
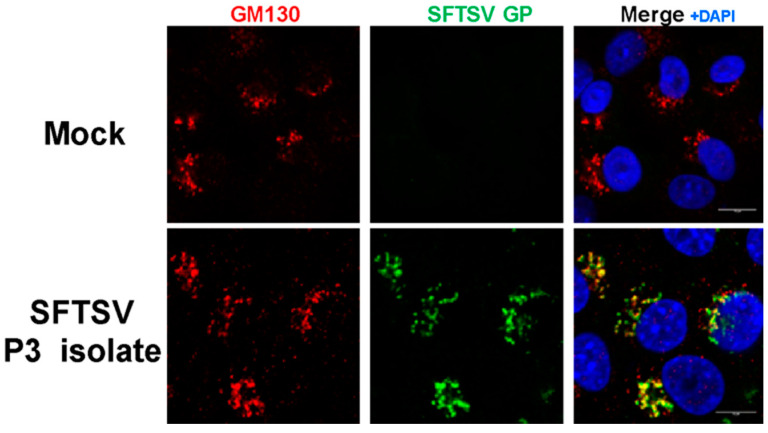
SFTSV viral particles co-located with the Golgi apparatus in Vero E6 cells. GM130 (red) was used as a *cis*-Golgi marker (BD, #610822). SFTSV GP (green) antibody was created against the SFTSV HB29 strain (Abnova, #PAB27171). DAPI (blue) was used to stain nuclei. Cells were infected with SFTS virus at a multiplicity of infection of 4 and incubated for 5 days.

The expression of the SFTSV GP was investigated by immunofluorescence microscopy to confirm SFTSV infection in Vero E6 cells. Viral GP was detected in the cytoplasm of infected cells and localized within the Golgi apparatus using SFTSV serum-specific pAb (Figure 2). These data are consistent with previous reports of intracellular SFTSV localization [2,47,48].

**Figure 3:**
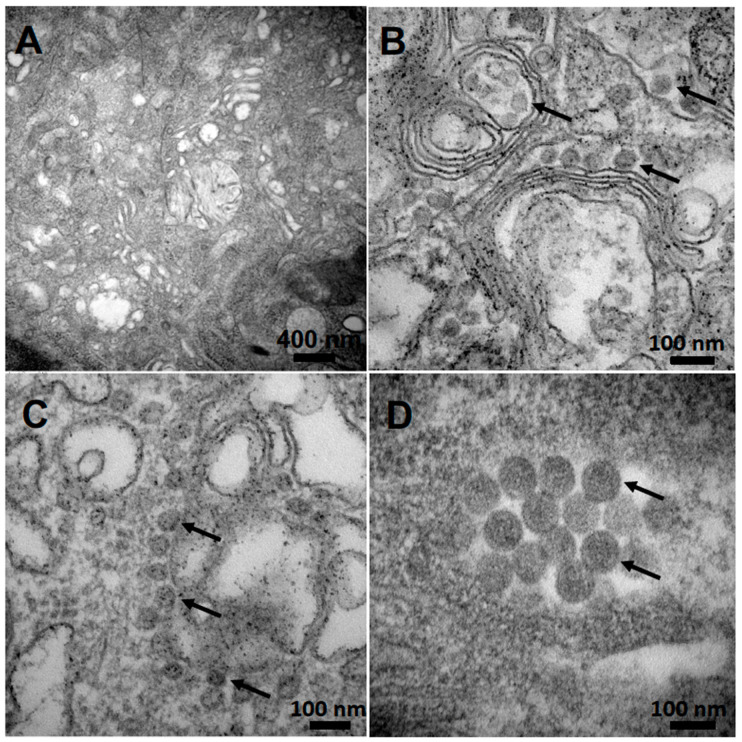
TEM images of Vero E6 cells infected with SFTSV from Passage 3. (**A**) uninfected controls; (**B**,**C**) cells with the viral particles (indicated by arrows) at day 3 post-infection; (**D**) magnified view (direct mag: 150,000×) of the viral particle structures (indicated by arrows) at day 6 post-infection.

To confirm the presence of complete virus particles in the SFTSV stock by TEM, Vero E6 cells were infected and subsequently processed for imaging at 3 d and 6 d after infection. Intact virus particles were seen throughout the cell at both 3 d and 6 d after infection and were absent in the uninfected controls (Figure 3A,B).

Virus particles had an approximate diameter of 100 nm (Figure 3D), were seen adjacent to membrane structures identified as the Golgi apparatus, and were often found in vesicle-like structures (Figure 3B,C). These observations are consistent with previous studies [2,47,48] which demonstrate that SFTSV structural proteins are localized in the ERGIC compartment and Golgi complex of infected cells, suggesting that these viral components start to assemble in those cellular compartments and that the viral glycoproteins are required for transporting the RdRp and the NP proteins to the ERGIC and Golgi complex.

### 3.3. Target-Enrichment Whole-Genome Sequencing

Full-length genomic sequences were obtained from the SFTS case (SFTSV strain AFMC 17-1), the RT-qPCR-positive tick pool (SFTSV strain USAMRIID-HLP23), and the Passage 3 isolate from Vero E6 cells (SFTSV strain USAMRIID-HLP23_VE6). The three viruses had the same genome organization, with three genomic segments containing four main ORFs. The S segment (1746 nt) contained two ORFs of 882 and 738 nt in an ambisense organization, encoding two proteins of 293 and 245 amino acids, identified as the NSs and the N proteins, respectively. The M segment (3378 nt) contained one ORF encoding a protein of 1073 amino acids, identified as the GP precursor. The L segment (6368 nt) contained one ORF encoding of 2084 amino acids, identified as the L protein, with its characteristic RdRp domain.

Comparison of the entire S, M, and L segments between SFTSV strains AFMC 17-1, USAMRIID-HLP23 strain, and USAMRIID-HLP23_VE6 showed a high level of homology (>99%) both at the nucleotide and at the amino-acid level (Table 3).

All sequences were deposited into GenBank with the following accession numbers—SFTSV strain AFMC 17-1: MT683683, MT683684, and MT683685; USAMRIID-HLP23: MN395043, MN395044, and MN395045; USAMRIID-HLP23_VE6: MN450761, MN450762, and MN450763.

### 3.4. Phylogenetic Analysis

**Figure 4:**
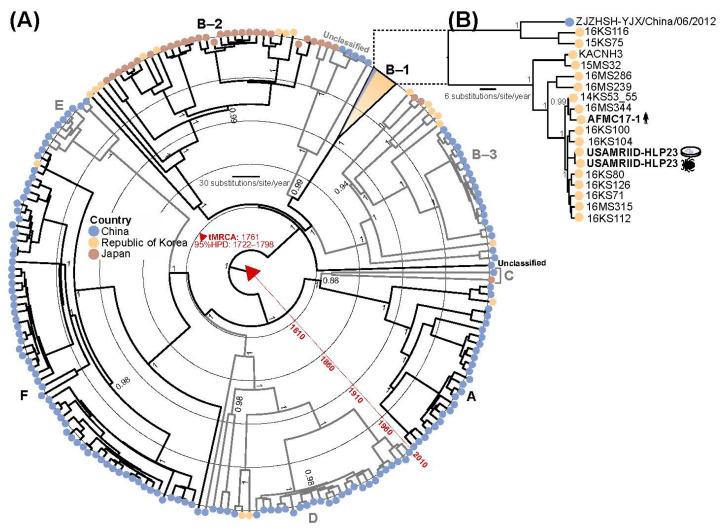
Radial maximum-clade credibility trees estimated using (**A**) 207 and (**B**) 19 B-1 genotype concatenated coding-complete SFTSV genomes. Circles at tree tips are colored by country of isolation. Time to the most recent common ancestor (tMRCA) and the 95% highest posterior densities (HPD) are shown. Posterior support >0.7 is displayed for major tree nodes. Strain names of sequences generated in this study are shown in bold along with host/source information and other B-1 genotype sequences. Tree branches are scaled by substitutions per site per year. The inset tree (**B**) contains recently published sequences from KOR that are not included in (**A**) [38].

Concatenated coding-sequence phylogenetic analyses using all ORFs were combined with publicly available and complete SFTSV genomes. We estimated that AMFC 17-1, tick pool, and Vero E6 isolates belong to the B-1 genotype (Figure 4 and Figure S1). AFMC-17 is most closely related to KOR strain 16MS344, whereas the tick-related strains were closely related to KOR strains 16KS100 and 16KS104. No geographical sampling data are publicly available of closely related strains [38].

### 3.5. Genomic Analysis

**Figure 5:**
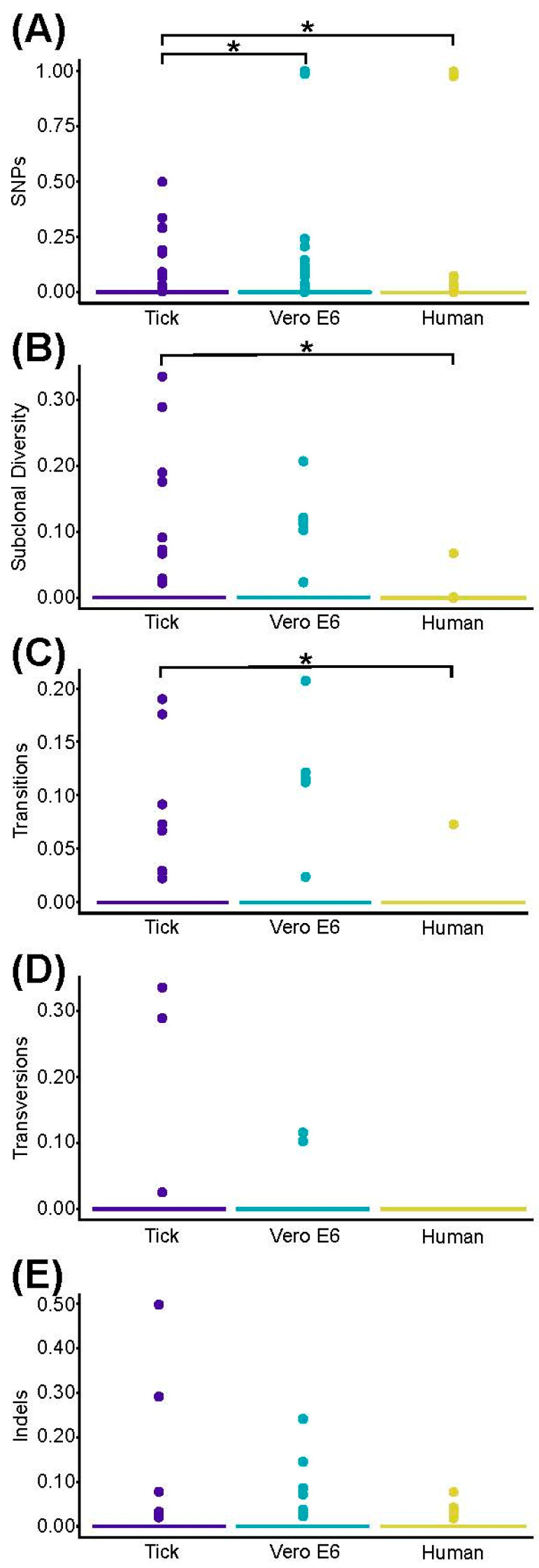
Viral diversity analysis of the SFTSV populations from AMFC 17-1 (human), USAMRIID-HLP23 (tick pool), and USAMRIID-HLP23_VE6 (Vero E6) isolates. For each sample, the three genomic segments (L, M, and S) were concatenated and the frequencies of each parameter for every nucleotide position with at least 100 reads populated. For every parameter and each sample, median frequencies were calculated along the entire genome. Error bars (representing the inter-quartile range) are not visible due to the *γ*-axis scale, determined by the presence of outliers. Outliers are represented by colored dots. (**A**) Frequency of SNPs (nucleotide positions presenting with a base change that is supported by at least 2% of the reads and by no more than 50% of those). (**B**) Subclonal diversity (SCD: events—SNPs and indels—per site per genome). (**C**,**D**,**E**) Individual frequencies (events per site per genome) of transitions, transversions, and indels (insertions and/or deletions), respectively. An asterisk indicates that differences were significant (*p* < 0.05) using the Wilcoxon rank sum test (Table S2).

The genomic diversity of the viral populations was compared among the human SFTSV isolate, the tick pool isolate, and the viral isolate from the Vero E6 cells, using as a reference the consensus sequence of the virus from the ticks. Raw data from the VSALIGN analysis pipeline [37] representing the frequency of each parameter across the entire genome (subclonal diversity, and frequency of SNPs, transitions, transversions, and indels) is depicted in Table S2. Per-sample averages and medians with standard deviations and interquartile ranges for each parameter across the entire genome (genome coverage, depth of coverage, subclonal diversity, and frequency of SNPs, transitions, transversions, and indels) are summarized in Table S3. A quasi-complete level of genome coverage (range 81.4–100%) along with a high depth of coverage (range 4095.1–45,539.7 reads) for every sample was obtained (Table S3). The frequency of SNPs was slightly lower in the viral isolate from the Vero E6 cells when compared to the tick pool (*p* = 0.028; Figure 5).

Although the average levels were also slightly lower in the Vero E6 viral isolate, these differences were not significant for SCD or for the frequency of transitions, transversions, and indels (Figure 5). When comparing the virus populations from the tick and the human SFTS case, all parameters presented with lower than average levels in the human SFTS case. These differences were significant for the frequency of SNPs, the SCD, and the frequency of transitions (*p* = 3.989 × 10^−5^, *p* = 0.013, and *p* = 0.0036, respectively), but not for the frequency of transversions and indels (*p* = 0.052 and *p* = 0.456, respectively) (Figure 5).

Mutations (consensus nucleotide changes supported by >50% of the reads) along the entire genome of the viral isolate from the Vero E6 and from the human case were populated with the consensus sequence from the tick isolate as the reference. The genome of the viral isolate from Vero E6 cells had three mutations along the entire genome: two in the L segment (L1287 T to C and L6275 A to T) and one in the S segment (S1455 T to C) (Table 4). The M segment sequence had no mutations. Two of these mutations (L1287 and S1455) were already present among the viral subpopulations from the tick pool as minor variants (2.6% and 19.9% of the reads, respectively). The L1287 nucleotide change resulted in an F→S amino acid residue change at position 424 of L’s RdRp domain. The second nucleotide change in this genomic segment (L6275) was located outside of the coding sequence (CDS). The nucleotide change in the S segment (S1455) was a silent mutation that did not result in any amino acid changes. The virus from the SFTS case contained 18 mutations along the entire genome, including six that resulted in amino acid changes: two in L’s RdRp domain, three in GP, and one in NS. The remaining 12 nucleotide changes were silent mutations.

### 3.6. Lethality in IFNAR^-/-^ Mice

**Figure 6:**
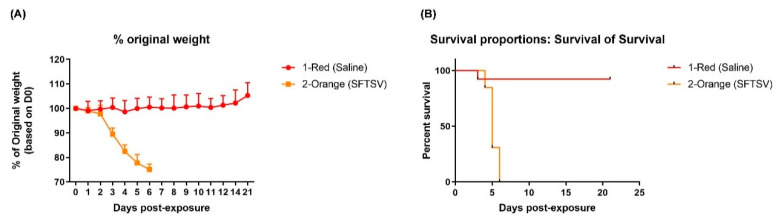
SFTSV lethality in interferon (α/β) receptor knockout (IFNAR^-/-^) mice. (**A**) Weight curve from the SFTSV-infected (orange) and control (red) mice. (**B**) Survival curves for mice exposed to SFTSV (orange, experimental group) versus inoculated with saline solution (red, control group). Thirteen animals per group (6 male and 7 female) were used.

SFTSV strain USAMRIID-HLP23_VE6 was used to evaluate the lethality of the virus in vivo. Interferon (α/β) receptor knockout (IFNAR^-/-^) laboratory mice were divided into two groups of 13 animals per group. The control group was inoculated with saline solution whereas the experimental group was inoculated subcutaneously with 1 × 10^5^ TCID_50_ SFTSV. Among the animals infected with SFTSV, the most common clinical signs of infection that were observed were subdued, ruffled fur, hunched posture, weight loss, decreased mobility, reluctance to move. Control animals maintained weight during the entire experimental period (21 d), whereas the experimental group began to drop weight by post-inoculation Day 3 (Figure 6A). Only one animal from the control group died, on Day 3, for unknown reasons. In contrast, animals from the experimental group were euthanized starting on Day 5 as they reached endpoint criteria for euthanasia. Animals were euthanized based on a combined scoring threshold for several parameters including weight loss, appearance and activity. Animals scored most frequently based on appearance, activity and weight loss. No animal in the experimental group survived beyond post-inoculation Day 6 (Figure 6B). There were no observable differences in disease severity between male and female mice.

## 4. Discussion

Syndromic and environmental surveillance resulted in the detection of SFTSV in a pool of ticks collected at a ROKA training area in KOR and in an SFTSV-infected ROKA soldier assigned to the same ROKA base. The complete characterization of both specimens using advanced genomic tools enabled the production of a high-titer viral exposure stock that, due to its detailed characterization and accurate documentation, will be useful for the development of animal models for SFTS that fulfill the requirements of the main regulatory agencies.

The virus was amplified and isolated in cell culture producing a high-titer viral stock, as determined by titration through a multiplex RT-qPCR-based TCID_50_ assay. TEM analysis and immunofluorescence microscopy demonstrated clear virion-like particles and SFTSV properties consistent with previous studies [2,47,48].

Viral genomes from the tick specimens (USAMRIID-HLP23), Vero E6 cells (USAMRIID-HLP23_VE6), and the human specimen (AMFC 17-1) were deeply characterized by using target-enrichment whole-genome sequencing. Both maximum-likelihood and Bayesian approaches estimated a close phylogenetic relationship between human and tick sequences. The overlapping time and geographical location of sample isolations suggest that the soldier may have been infected by a strain closely related to USAMRIID-HLP23. However, additional geographical information of closely related strains is needed to support this claim [38].

The phylogenetic analysis also demonstrated that virus isolation and propagation in Vero E6 cells did not introduce substantial genetic changes in the original tick virus. Results of the deep-sequence population genomic analysis showed very little genomic diversity among the viral populations of the tick virus and the cell-culture isolate. All parameters remained low and at comparable levels in the Vero E6 virus isolate compared to the SFTSV detected in the pool of ticks, with the exception of the frequency of SNPs that were slightly lower. Furthermore, only three nucleotide changes appeared in the cell-culture isolate along the entire genome, only one of which resulted in an amino acid change affecting L’s RdRp domain. This mutation, along with the nucleotide change in the nucleocapsid protein, was already present among the SFTSV subpopulations from the pool of ticks as minor variants that were further selected and becoming a dominant variant in the cell-culture isolate. No changes were observed in the GP precursor (M segment). A certain degree of genetic variation is expected as a result of the adaptation of the virus to the cell line in which it is grown, and similar or higher levels are usually observed in other commonly used cell-culture viral isolates [49].

The combined results of the phylogenetic and the genomic analysis demonstrates that (a) the SFTSV viral stock USAMRIID-HLP23_VE6 is highly representative of the natural viral strain reportedly transmitted by ticks and (b) both SFTSV variants are very closely related to the SFTSV strain that was the causative agent of SFTS in the ROKA soldier, with the aforementioned SFTSV detected in the pool of ticks very likely constituting the source of this infection. Furthermore, the use of the viral stock USAMRIID-HLP23_VE6 to model SFTS disease was tested in IFNAR^-/-^ laboratory mice, with the virus group demonstrating high lethality (100% of animals either reaching euthanasia criteria or succumbing to disease) versus the mock group. This lethality is comparable to that achieved with a similar mouse strain subcutaneously exposed to 10^6^ TCID_50_ of human-derived Japanese SFTSV strain SPL010 [50]. In both cases, animals succumbed before post-exposure Day 7, confirming that the tick-derived SFTSV USAMRIID-HLP23_VE6 and the human-derived isolate behave similarly in laboratory mice. Altogether, the important features discussed above highlight the value of SFTSV USAMRIID-HLP23_VE6 as a well-characterized starting material to model SFTSV in animals.

Overall, the work performed in this study illustrates a comprehensive process for the rapid characterization and subsequent production of well-characterized quality viral stocks. These can be used as reliable and reproducible controls to study the biological aspects of viral agents while providing valuable information during the development of medical countermeasures, such as vaccines and therapeutic drugs.

## Figures and Tables

**Table 1:** Total number of tick specimens and pools distributed by species and lifecycle stage and analyzed for the presence of SFTSV by RT-qPCR.

Taxonomy		Larvae	Nymph	Adults	Total
Asian longhorned ticks (*Haemaphysalis longicornis* Neumann, 1901)		4776	8214	57	13,047
*Haemaphysalis flava* Neumann, 1897		68	250	78	396
*Ixodes nipponensis* Kitaoka and Saito, 1967		0	10	130	140
*Haemaphysalis phasiana* Saito, Hoogstraal and Wassef, 1974		17	35	0	52
Brown dog tick (*Rhipicephalus sanguineus* (Latreille, 1806))		0	0	1	1
	Total specimens	4861	8509	266	13,636
	Pools	186	445	95	726

**Table 2:** Primer and probe sequences used in the quantitative SFTSV multiplex RT-qPCR assay.

Genome Segment	Primer	Primer/Probe Sequence 5′-3′
L	L-F-3-mod	AGTCTAGGTCATCTGAYCCGTTYAG
	L-R-3	TGTAAGTTCGCCCTTTGTCCAT
	L-Probe-3-mod	[HEX] CAATGACAGAYGCCTTCCATGGTAATAGGG [BHQ1]
M	M-F-3-mod	AAG AAR TGG YTG TTC ATC ATT ATT G
	M-R-3-mod	GCC TTR AGR ACA TTG GTG AGY A
	M-Probe-3-mod	[FAM] TCA TCC TCC TTG GRT ATG CAG GCC TCA [BHQ1]
S	S-F-3-mod	GGRTCCCTGAAGGAGTTRTAAA
	S-R-3-mod	TGCCTTCACCAAGACYATCAATGT
	S-Probe-3-mod	[TexasRed] YTCTGTCTTGCTRGCTCCRCGC [BHQ-2]

**Table 3:** Comparison of the complete genomic sequences of SFTSV detected in the SFTS case, the tick pool, and the Vero E6 isolate, showing a high level of both nucleotide and amino-acid homology. Data are presented as a percentage-of-identity matrices, with the number of changes indicated between parentheses, elaborated with either nucleotide (upper right half) or amino acid (lower left half) sequence alignments of full L, M, and S genomic segments.

	Strains	AMFC 17-1	USAMRIID-HLP23	USAMRIID-HLP23_VE6
L segment	AMFC 17-1	-	99.89 (7)	99.92 (5)
	USAMRIID-HLP23	99.9 (2)	-	99.97 (2)
	USAMRIID-HLP23_VE6	99.95 (1)	99.95 (1)	-
M segment	AMFC 17-1	-	99.79 (7)	99.79 (7)
	USAMRIID-HLP23	99.72 (3)	-	100
	USAMRIID-HLP23_VE6	99.72 (3)	100	-
S segment	AMFC 17-1	-	99.77 (4)	99.83 (3)
	USAMRIID-HLP23	99.81 (1)	-	99.94 (1)
	USAMRIID-HLP23_VE6	99.81 (1)	100	-

**Table 4:** Mutations (positions with nucleotide changes supported by >50% of the reads) in the SFTSV populations from the Vero E6 isolate and from the SFTS case in relation to the virus from the ticks. Position nt/Position aa: nucleotide/amino acid position in the virus from the ticks; RdRp: RNA-directed RNA polymerase; GP: glycoprotein; NS: nonstructural protein; NP: nucleocapsid protein; NC: non-coding; Freq.: percentage of reads supporting the corresponding nucleotide; Codon: amino acid residue and the corresponding codon. Bases with and asterisk (*) in the virus from the ticks were supported by less than 100 reads, and the values are shown for comparison purposes. Nucleotide and amino acid mutations in the Vero E6 isolate and in the virus from the SFTS case are shown in bold.

							Consensus Changes					
Position nt	Segment	Position aa	Protein	Tick			Vero E6			Human		
				Base	Freq.	Codon	Base	Freq.	Codon	Base	Freq.	Codon
1287	L	424	L	T	97.4	F (TTT)	C	98.6	S (TCT)	C	99.7	S (TCT)
2329		771		T	100	Y (TAT)	T	99.9	Y (TAT)	C	99.8	Y (TAC)
3070		1018		A	100	I (ATA)	A	99.9	I (ATA)	T	99.9	I (ATT)
3574		1186		C *	100 *	H (CAC)	C	99.9	H (CAC)	T	99.9	H (CAT)
4170		1385		G	92.7	R (AGA)	G	88.8	R (AGA)	A	99.9	K (AAA)
6088		2024		A	100	A (GCA)	A	99.9	A (GCA)	G	99.6	A (GCG)
6275		NC	NC	A	98.1	NC	T	99.8	NC	T	99.9	NC
40	M	8	GP	A *	100 *	T (ACC)	A	99.9	T (ACC)	T	100	S (TCC)
63		15		T	100	I (ATT)	T	99.9	I (ATT)	C	100	I (ATC)
564		182		T	100	P (CCT)	T	99.9	P (CCT)	C	99.6	P (CCC)
573		185		T	100	P (CCT)	T	99.9	P (CCT)	C	97.8	P (CCC)
1291		425		T	66.4	L (TTG)	T	88.4	L (TTG)	A	99.9	M (ATG)
1951		645		G *	100 *	A (GCA)	G	99.9	A (GCA)	A	99.9	T (ACA)
3237		1073		A	100	A (GCA)	A	99.9	A (GCA)	T	99.9	A (GCT)
280	S	84	NS	G	97.1	C (TGC)	G	88.4	C (TGC)	A	99.9	Y (TAC)
716		229		T	100	D (GAT)	T	99.9	D (GAT)	C	99.9	D (GAC)
860		277		C	100	T (ACC)	C	99.9	T (ACC)	A	100	T (ACA)
1455		83	NP	T	80.1	L (TTA)	C	99.9	L (TTG)	C	99.8	L (TTG)

## Supplementary Materials

The following are available online at https://www.mdpi.com/1999-4915/12/9/958/s1. Figure S1: Maximum-likelihood phylogenetic tree based on coding-complete genomes of the SFTSV strains characterized in this study (tick, Vero E6 isolate, and human) and all publicly available SFTSV coding-complete genomes from GenBank. Table S1: Accession numbers and associated metadata of the SFTSV genomic sequences from GenBank included in the phylogenetic analysis and in the haplotype network. Table S2: Results of the genomic diversity analysis of the viral populations from the human SFTSV isolate, the tick pool isolate, and the isolate from Vero E6 cells: subclonal diversity and frequency of SNPs, transitions, transversions, and indels across the entire genome. Table S3: Results of the genomic diversity analysis of the viral populations from the human SFTSV isolate, the tick pool isolate, and the isolate from Vero E6 cells: per sample averages and medians for genome coverage, depth of coverage, subclonal diversity, and the frequency of SNPs, transitions, transversions, and indels across the entire genome.
