# Supplementary figures and images for: A Model for the Production of Regulatory Grade Viral Hemorrhagic Fever Exposure Stocks: From Field Surveillance to Advanced Characterization of SFTSV
Source: Viruses. 2020 Aug 29;12(9):958. doi: 10.3390/v12090958 (PMC7552075; doi:10.3390/v12090958)

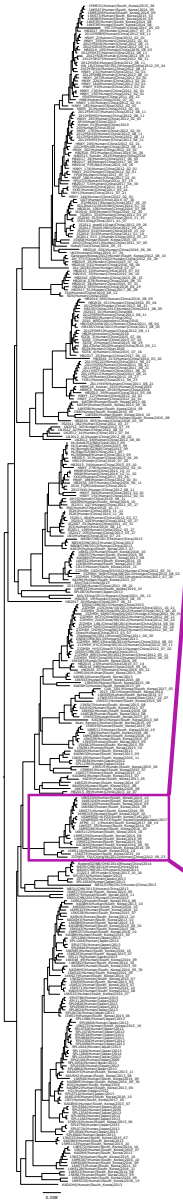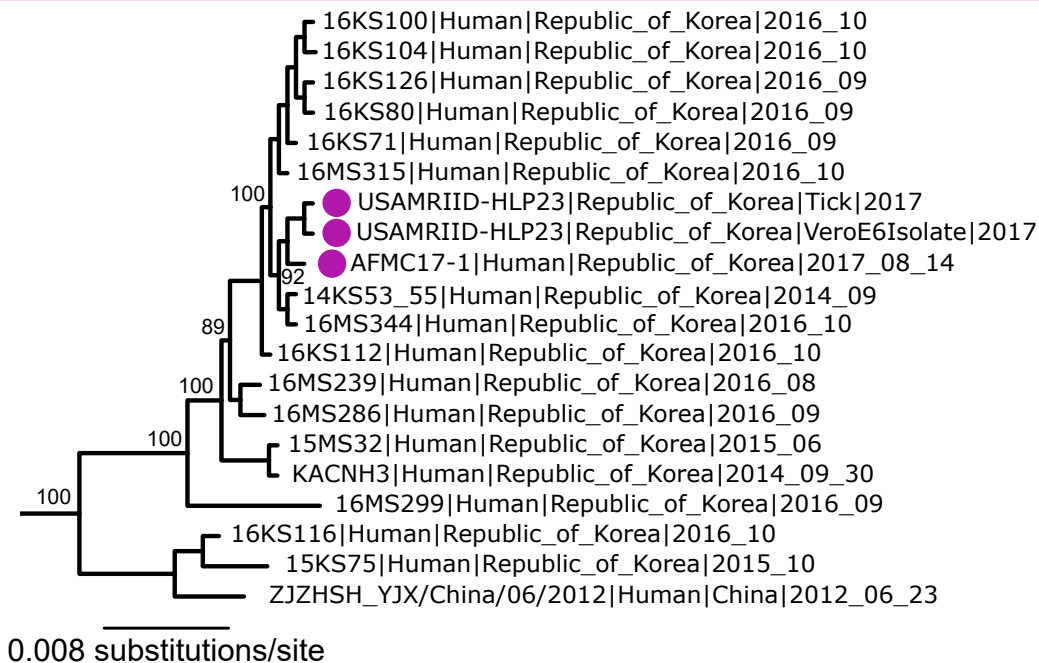

Supplement: Supplementary file 1 [file viruses-12-00958-s001.zip › FigureS1.pdf]
